# Sexual Risk Behaviors, HIV Prevalence and Access to Reproductive Health Services Among Young Women Migrant Workers in the Industrial Zones in Vietnam

**DOI:** 10.3389/frph.2021.775375

**Published:** 2021-12-03

**Authors:** Toan Ha, Stephen L. Schensul, Jean J. Schensul, Trang Nguyen, Nam Nguyen

**Affiliations:** ^1^Department of Infectious Diseases and Virology, Graduate School of Public Health, University of Pittsburgh, Pittsburgh, PA, United States; ^2^Department of Public Health Sciences, School of Medicine, University of Connecticut, Farmington, CT, United States; ^3^Institute for Community Research, Hartford, CT, United States; ^4^Institute of Social and Medical Studies, Hanoi, Vietnam

**Keywords:** sexual risk behavior, HIV, women migrant worker, industrial zone, Vietnam

## Abstract

**Background:** Young migrant workers working in the industrial zones (IZ) in low and middle-income countries are at risk for HIV and other sexually transmitted diseases. This study examines the sex-related risks of young women migrant workers in the IZ in Vietnam.

**Materials and Methods:** This cross-sectional survey was conducted among 1,061 young migrant women working in the IZ park in Hanoi, Vietnam. Multivariate logistic regression analysis was used to identify factors associated with HIV testing and condom use at last sex.

**Results:** A total of 1,061 young women migrant workers completed the survey in which 652 participants consented to take the initial rapid HIV test. All but one participant tested negative indicating a HIV prevalence of 150 (95% CI: 27–860) per 100,000 population among this population. There were no differences in sexual behavior, use of sexual and reproductive health services, HIV knowledge, perceived HIV risk or alcohol use between those who were HIV tested and those not tested. Single participants reported high rates of first sex while living in the IZ and high rates of condom use during the first-time sex, however, they had low levels of condom use at last sex. While the majority of married participants used the SRH/HIV services, nearly 80% of the single participants who reported having sex never used SRH/HIV services since living in the IZ. However, single participants were over 4 times more likely to use condoms at last sex compared to married participants (OR = 4.67; 95%CI = 2.96–7.85). Participants with vocational school or higher education was more likely to use condom (OR = 2.19; 95%CI = 1.05–4.57). Neither HIV knowledge or alcohol use were associated with condom use.

**Conclusions:** Although HIV prevalence is very low among young women workers in the IZ in Vietnam, a significant number of them engaged in risky sexual behavior and low levels of condom use at last sex as well as low level of using SRH/HIV services highlights a need to develop interventions that provide tailored-made and cultural appropriate SRH education for unmarried female migrant workers to prevent risky sexual behaviors, sexually transmitted diseases and unwanted pregnancy.

## Introduction

Over the last three decades, industrial zones (IZ) in low and middle-income countries (LMICs) have rapidly expanded as global corporations search for the lowest unit cost for assembly of piece goods for high-income country markets. Global brands or their production intermediaries are attracted to a country because of tax breaks on importing of components, country-provided infrastructure (e.g., roads, buildings, proximity to ports) and a relatively well-educated but underemployed rural workforce willing to migrate from rural villages to peri-urban areas for available jobs. Worldwide, young women <30 years of age constitute 50–76% of the workers in global IZ workforce (1, 2). These young women workers are favored because they are perceived to be more efficient than men at repetitive tasks, more willing to accept difficult or uncomfortable working conditions.

Prior research has generated substantial evidence to support the view that young IZ women workers are at risk for HIV (3, 4). Studies in Asian countries including China, India, Philippines, Laos, and other countries show higher HIV rates among female migrant workers than in the general population. A systematic review of studies among rural to urban migrants in China found that HIV prevalence among female migrants was 12 times higher as compared to the general population (3). Studies examining HIV risk among both male and female migrant workers in Asian countries including Vietnam (5), Thailand (6), China (7–11), India (12, 13), and Bangladesh (14) have shown that migrants are more likely to engage in HIV and risky sexual behaviors (5, 14–17), less likely to use condoms at first sex (8, 18), and at last sexual encounter (5) and do not consider themselves to be at risk of HIV when having unprotected sex (7, 17).

Factors related to sexual risk behaviors among Asian migrant workers include low self-efficacy (6, 8), low levels of education (14, 19), separation from family, new and unfamiliar urban behavioral norms (20), perceived peer norms (6) poor knowledge of HIV (6, 8, 10), limited access to health care (14, 19) and exploitative working conditions that include sexual violence and harassment (21, 22). Living away from families and familiar socio-cultural norms can result in loneliness that leads some migrants to seek companionship and sexual intimacy, leading to HIV infection (23), STIs and unintended pregnancies (24). Due to economic pressures some migrant women rely on commercial sex to supplement their income, increasing their HIV risk (25). Studies have found that the high cost of healthcare may present barriers for migrant workers such as limiting their access to HIV/STI testing and treatment services (26). Migrants may use increased alcohol and other drugs to offset the stressors of migrant life, and feelings of depression and anxiety (22, 27). Migrants are vulnerable to HIV because of exploitative working conditions, including sexual violence (21) and because of the need for additional income to support their families. Poor working conditions, limited legal rights, and changing social contexts may expose migrant workers to health risks and hinder their access to health care services (21, 28).

Despite the large amount of literature on sexual risks among migrants in Asia, and especially among women migrants to urban industrial or employment zones (8, 13, 16, 29), there is paucity of research currently on HIV prevalence and related sexual risk exposure among women in the industrial zones of Vietnam. Hundreds of thousands of rural women migrate to the larger and smaller cities of Vietnam, to work in industrial zones. Little is known about their adaptation to living away from their families, and their exposure to sexual and other health risks including HIV. This paper attempts to fill this gap through an examination of the HIV prevalence, sexual risk exposure and access to reproductive health services among Vietnamese young women who migrate to the IZs on the outskirts of Hanoi.

In 1986 Vietnam adopted a liberalized economic model, and as a result of foreign investment, generated rapid industrialization of the country's major cities. An important component of that industrialization was the IZs. Vietnam currently has 257 operational IZs and ~3.6 million workers of whom 60% are women; of these women 70.0% are below the age of 29 (30, 31). Studies in Hanoi and Ho Chi Minh city, describe poor living conditions among IZ workers who often rent cheap dwellings called “rent clusters” in slums with no outlet for wastewater and sewage overspills and poor ventilation (32, 33). In Vietnam, a further complication is the *ho khau* system in which individuals can only access government services in their rural communities of origin, requiring IZ workers who are far from their natal families to pay for health and other services when they are in the city (34). Sexual and reproductive health (SRH) services mainly target married couples (35), which combined with limited income, results in IZ women workers seeking health care services only for extreme need. A study among IZ women workers in Vietnam reported that only 10.2% of respondents have accurate knowledge about safe sex (36). The increase in pregnancies and abortions among IZ female workers have been documented (37) and studies among women workers in IZs reported misconceptions about HIV/STIs (38). These limited data on women in IZs suggests the high potential for sex-related risks, HIV exposure and infection in this population.

Despite a decrease in overall HIV prevalence in Vietnam (0.3%), HIV remains a concern with an estimated 5,200 new HIV infections and 5,000 AIDS-related deaths in 2020 (39). Currently, most cases are concentrated among injecting drug users (IDUs), men who have sex with men (MSM) and female commercial sex workers. Although IZ workers are also a concern, no research exists yet on HIV prevalence among workers, especially female workers in Vietnam's IZs. The primary objective of this paper is to explore the sex-related risks of women IZ workers, aged 18–29. Based on ample evidence from other LMICs, we hypothesized that young women migrant workers would have a higher level of sex-related risk exposure, a greater prevalence of HIV and less access to reproductive health services than the general population of women in the target age group in Vietnam. We also hypothesized that subgroups, particularly single women and married women living without their husbands would be exposed to greater sex-related risk, and significantly less engagement in reproductive health care and prevention.

## Materials and Methods

### Study Site and Participants

A cross-sectional study was conducted with 1,061 young migrant women from January, 2019, to November, 2020, in Thang Long Industrial Park, located in Dong Anh district on the outskirts of Hanoi, the capital of Vietnam. The Park currently has 31 major industries focusing on assembly of consumer goods, electronic components, vehicle accessories, automotive electronics and civil mechanical appliances (40). It is estimated that there are over 60,194 workers working in the Park (41). The majority of the young women workers live in “rent clusters” which are privately owned and developed by landlords to accommodate the flow of workers seeking residences in the surrounding areas of the Park. A minority of workers, particularly those who are just beginning their work in the IZ live in dormitories provided by their factories.

Eligibility criteria for participation in the study included: (1) female, (2) between 18 and 29 years of age; (3) single, currently married but living separately from husband or partners, or living alone (separated, divorced or widowed), (4) Six or more months of work experience in the IZ; (5) migrated from a rural area or another province prior to IZ employment.

### Mapping and Selection of Participants

#### Sample Size

Cluster designs that include people living/working in close proximity (such as women living together in a rent cluster or dormitory) can affect the power of a study due to the interactive effect of similar-behaving subjects. Our sample size determination accounted for the cluster design through or intra-class correlation (ICC) within the primary sampling unit: rent clusters and dormitories. We accounted for ICC by basing power on the effective sample size (42). Our sampling approach involved collecting data from 419 rent clusters and two dormitories for a total of 1,061 respondents.

#### Sampling Process

A multistage, clustered sampling method was used to select women workers in the IZ. First, 779 rent clusters and two dormitories located near the IZ were mapped and enumerated. Only rent clusters, with six or more eligible participants were selected that resulted in a sample of 419 eligible rent clusters, and 360 ineligible rent clusters. All female workers aged 18–29 were identified and listed in each eligible rent cluster. Those listed were numbered from the youngest to the oldest in each rent cluster. Based on the developed list, the sampling interval (k) was obtained by dividing the total of eligible participants by 6. A random number (x) between 1 and the sampling interval (k) was chosen as the starting point by using a table of random numbers. A total of 936 participants were selected from the 419 rent clusters. Of the total of 175 eligible residents identified and listed by the dormitory managers in two selected dormitories, 125 participants were randomly selected for an overall total of 1,061 young women workers to be interviewed.

### Survey Interview

Eligible women received a face-to-face anonymous interview using structured questionnaires conducted by trained field researchers in a private setting. All interviewers received training in interviewing techniques, developing rapport, ensuring confidentiality, and answering questions raised by participants. Participants received USD $3.0 in compensation for participating in the survey interview and USD 1.5 for getting an initial HIV test. Eligible participants were invited to an after-hours meeting in a nearby commune health center organized by the research team. At the meeting, they were fully informed about the study, the activities in which they were expected to participate, topics to be covered by the survey, the amount of time required for the interview, their right to refuse or to withdraw, and the procedures for ensuring confidentiality of interviews. A research team member then handed the participant a written informed consent form and asked her to read it along. Any questions a participant had were answered by the research team members. When there were no further questions the participant was given time to consider the risks and benefits of participation. Once agreeing to join the study, she was asked for written informed consent after which she was enrolled.

### HIV Testing

The HIV testing protocol required three tests, two for screening and diagnosis of HIV and the third for confirmation of diagnosis of HIV and initiation of ART. To obtain HIV testing consent, project field researchers conducting the survey asked respondents if they would be willing to take an HIV test. They were assured that opting out of the test would not result in any negative consequences. If they consented, the field researcher returned with a trained health worker who provided pre-test counseling and performed a finger-prick blood-based HIV test. The SD Bioline HIV-1/2 3.0 test (Standard Diagnostics, South Korea), was first administered to the participant; if this test was negative, no further additional testing was conducted and the health care worker provided post-test counseling encouraging approaches to reducing sexual risk. However, if a sample was positive (reactive) for HIV, the initial test was followed up with a second test, the Alere Determine HIV ½ (Abbott Laboratories, United Kingdom). For a negative test, participants were given counseling and no further confirmation testing. For a positive test, the participant was informed about the results, given counseling and was accompanied by the project staff to the nearby Dong Anh District Health Center which has a Ministry of Health-certified district-level confirmatory laboratory for the third, HIV confirmation, test. The study protocol required that individuals with a third confirmed HIV-positive result be resulted in counseling and referral to a clinic for initiation of ART treatment and care.

#### Sociodemographic Variables

The sociodemographic variables in the survey included age, income, education, ethnicity, origin of residence, relationship status, health insurance and young women workers' living conditions such as house status, working hours, income and expenditure.

#### Sexual Behavior/Sex Risk

Participants were asked by whether they had ever had sexual intercourse (yes/no). Those who reported ever having had sex were asked about age at first sex, with whom (e.g., boyfriend, husband, etc.), where (e.g., at the home village or IZ), whether they had engaged in sexual activities during the last 6 months (yes/no responses), number of sexual partners in the past 6 months, and whether they used a condom in their first sexual encounter and during the last 6 months preceding the interview (yes/no responses).

#### HIV Knowledge

HIV knowledge was measured with 15 yes/no/don't know questions. This included a screening question asking the respondent had ever heard of HIV/AIDS. Respondents who gave a negative response were excluded from the follow-up questions. Follow-up questions included one item on HIV transmission, three items on the prevention of HIV, and three items about HIV misconceptions concerning mosquito bites, sharing food with an HIV-infected individual and knowing that a healthy-looking person can have HIV/AIDS. Each correct answer was given one point, with a total score ranging from 0 to 15 point. A higher score indicates greater HIV knowledge (Cronbach alpha = 0.55).

#### Perceived Risk of HIV

Perceived risk of HIV infection was measured using two question asking participants' perception about the possibility that they and their sexual partner (for those who had one or more) would acquire HIV. The response format was a 5-point scale ranging from extremely unlikely (1) to extremely likely (5), for a scale score range of 1–10.

#### Use of Sexual and Reproductive Health and HIV (SRH/HIV) Services

Participants were asked if they had used sexual and reproductive health SRH/HIV services since they began working in the IZ. If yes, they were further asked to respond yes or no to a list of SRH or HIV services they used (e.g., contraceptive methods, HIV test, pregnancy care and delivery, gynecological exam).

#### Alcohol Consumption

Alcohol consumption was measured using the 3-item Alcohol Use Disorders Identification (AUDIT-C) questionnaire assessing frequency of drinking, typical number of drinks consumed on a drinking day, and frequency of binge drinking (43). Responses to each item were scored from 0 to 4 with higher scores indicating a higher level of alcohol use. The total scores were then categorized as low risk (0–4), moderate risk (5–7), high risk (≥8).

### Ethical Approval

This study was reviewed and approved by the Institutional Review Boards of the University of Connecticut Health Center, USA and the Institute of Social and Medical Studies, Hanoi, Vietnam.

#### Data Analysis

Descriptive analyses illustrate socio-demographic characteristics of the study participants. Frequencies and percentages were computed for categorical variables and means (±Standard Deviation) for continuous variables. Chi-square and Fisher's exact test was used to compare the differences in characteristics of participants' sexual behaviors. Multivariate logistic regression analysis was used to identify factors associated with HIV testing and condom use at last sex controlling for demographic factors (age, education, marital status, ethnicity and place of residence). A *p*-value was considered significant at <0.05.

## Results

### Socio-Demographic Characteristics

Of the total of 1,061 young women workers who completed the survey, the mean age was 22.8 years ranging from 18 to 29 (Table 1). The majority of respondents had completed high school. Over two thirds of the respondents (*n* = 760) were single. Most of the participants lived in rent clusters. Respondents' years of working at the current IZ ranged from <1 year to over 5 years with two thirds in the 2 to 5 year category and 14% over 5 years. While most of the respondents worked 8 h or less a day, nearly 24% were working over 8 h a day. Mean income was US$294 per month, ranging from $195 to $652.

**Table 1:** Participant characteristics.

**Variables**	**Single** **(*n* = 760)**		**Married^a^** **(*n* = 301)**		**Total** **(*n* = 1,061)**	
	***n***	**%**	***n***	**%**	***n***	**%**
**Age** (years), Mean (SD)	21.9 (2.6)		26.3 (2.5)		23.2 (3.2)	
**Education**						
Under high school	43	5.7	66	21.9	109	10.3
High school	650	85.5	192	63.8	842	79.4
Above high school	67	8.8	43	14.3	110	10.4
**Ethnicity**						
Kinh (the majority)	529	69.9	206	68.4	735	69.3
Other minority groups	231	30.4	95	31.6	326	30.7
**Residence**						
Rent cluster	695	91.4	241	80.1	936	88.2
Dormitory	65	8.6	60	19.9	125	11.8
**Years of working in the IZ**						
≤1 year	389	52.2	94	31.3	483	51.7
2–5 years	329	43.3	100	33.2	429	31.4
>5 years	42	5.5	107	35.5	149	17.2
**Working hours per day**						
8 h	587	77.2	223	74.1	810	76.3
>8 h	173	22.8	78	25.9	251	23.7
**Monthly income** (USD) Mean (SD)	287.8	52.5	301.9	64.5	291.7	56.8

a *Married including those who were currently married but not living with husband while working in the IZ or separated, divorced or widowed*.

### HIV Testing and HIV Prevalence

Of the total of 1,061 respondents, 652 (61.5%) consented to take the initial rapid HIV test (Table 2). Of those tested, only one woman was HIV positive. The remainder were HIV negative, indicating a prevalence rate of 150 (95% CI: 27–860) per 100,000 population of young women workers (aged 18–29).

**Table 2:** Multivariate analysis of factors associated with participants' consenting HIV testing.

**Characteristics**	**aOR**	**95% CI**	***P***
**Age**			
18–24	1		<0.001
25–29	0.39	0.28–0.72	
**Education**			
<=Secondary	1		
High school	0.93	0.51–1.62	0.637
Vocational and higher	1.06	0.45–2.35	0.745
**Marital status**			
Single	1		
Married	0.97	0.43–1.77	0.534
**Ethnicity**			
Kinh (the majority)			0.634
Other minority groups	0.93	0.45–1.37	
Residence type
Dormitory	1		<0.001
Rent cluster	9.45	3.94–28.50	
**Years of working in the IZ**			
>1 year	1		
>1–5 years	0.64	0.29–1.48	0.053
>5 year	0.24	0.12–0.72	0.032
**Ever had sex**			
Yes	1		0.645
No	1.09	0.55–1.57	
**Using a condom at the first sex**			
No			0.07
Yes	1.37	0.97–2.23	
**Used the SRH/HIV**^**a**^ **service while living in the IZ**			
No	1		
Yes	0.67	0.38–1.83	0.821
**Knowledge of HIV/AIDS (mean)**	1.24	0.91–1.50	0.176
**Perceived HIV risk**	1.13	0.89–1.13	0.262
**Alcohol consumption**			
Low risk	1		
Moderate and high risk	1.091	0.54–1.73	0.458

a *SRH/HIV: Sexual and Reproductive Health/HIV*.

Almost half (48.5%) of respondents did not opt to test for HIV. We hypothesized that those who rejected the HIV test might show greater sexual risk. Thus, we examined differences between those who were tested and those not tested, by demographic characteristics, sexual behavior, HIV knowledge and use of SRH service variables (Table 2). The results showed that participants living in the rent cluster were more likely to take the rapid test (OR = 10.05, 95% CI: 4.01–27.49) than those living in the dormitories and that older participant were less likely to take the HIV test (OR = 0.34, 95% CI: 0.19–0.63). There were no differences in sexual behavior, use of SRH/HIV services, HIV knowledge, perceived HIV risk or alcohol use between two groups.

#### Sexual Risk Behaviors

Despite the low prevalence of HIV in those women who were tested, sexual risk behavior is present among women in the IZ (Table 3) regardless of whether or not they were tested. We hypothesized that single women were more likely to engage in risky sexual behavior with men seeking paid sexual partners than married women and therefore we explored factors associated with sexual risk behavior among single women (*n* = 760) and women married (*n* = 301). The results showed that single participants had a higher level of having first sex in the IZ than married participants.

**Table 3:** Sexual behaviors among those who have sex (*n* = 512).

	**Single** **(*n* = 215)**		**Ever married** (*n* = 297*)		**Total** **(*n* = 512)**	***P***
	***n***	**%**	***n***	**%**	**%**	
**Age at first sexual intercourse**						
Before 18 yrs	8	3.7	7	2.0	5.7	0.279
After 18 yrs	215	96.3	290	98.0	94.2	
**Partner of first sexual intercourse**						
Boyfriend	196	91.2	31	10.4	44.3	0.000
Fiancé	17	7.9	52	17.5	13.5	
Husband	0	0.0	214	72.1	41.8	
Other	2	0.9	0	0.0	0.4	
**Condom use at the first sex**	179	83.3	139	46.8	62.1	0.000
**Place of having first sex**						0.000
While living rural areas	87	29.5	208	70.5	57.6	
While living in the IZ	128	59.0	89	41.0	34.6	
**Have had sex in the last 6 months**	145	67.4	229	77.1	73.0	0.012
**Have had sex with men who are not husbands**	N/A	N/A	11	3.7		
**Frequency of condom use in the past 6 months**						
None	17	12.6	98	43.8	32.0	
Often/Sometimes	66	48.9	86	38.3	42.4	0.000
Always	52	38.5	40	17.9	25.6	
**Condom use at the last sex**						
No	46	21.4	160	53.9	40.2	0.000
Yes	169	78.6	137	46.1	59.8	
**Have had pre-marital sex**	N/A	N/A	127	42.8		
**Have ever used SRH/HIV services while living in the IZ**	153	20.1	215	71.4	34.7	0.000

* *Four married participants did not respond to question about having sex*.

#### Single Participants

Among 760 single participants, 28.3% (*n* = 215) reported ever having had sex (Table 3). Over 18% (*n* = 36) reported having first sex before 18 years old. Participants reported a higher level of first penetrative sex while living in the IZ than in their home village. Eighty-three percent reported using a condom in their first sexual intercourse.

Of those women who had sex, 67.4% (*n* = 145) reported having sex in the last 6 months and only 38.5% always used a condom. Almost all the women reported having one sexual partner at a time, with only three participants reporting having two sexual partners.

Nearly 80% (*n* = 607) of the single participants who reported having sex had never used SRH/HIV services since living in the IZ. Among those who used (SRH/HIV) services (20.1%), over 60% used the services for a gynecological exam, followed by counseling on SRH (46.0%) and HIV test (9.8%). Only 1.3% used the services for contraceptive methods. The remainder used private clinics (19.6%), private hospital (5.2%), and only few used commune health centers near the IZ (1.3%).

#### Married Participants

Among the married participants, 42.8% (*n* = 127) reported having sex before marriage, primarily with the men who would become their husbands, with the majority initiating sex in their home village (Table 3). Condom use at first sexual experience was 46.8%. Among those who reported having sex in the last 6 months (*n* = 247), only 17.9% always use a condom, and about 46 % (*n* = 137) used condoms during the last sex. There were 11 married participants (3.7%) who have had sex in the last 6 months with men who were not their husbands.

Among those who were married, 71.4% (*n* = 215) had used SRH/HIV services since living in the IZ. Over two thirds used the services for gynecological exam (67.4%), counseling on SRH (20.0%) and contraceptive methods (8.8%). A small number used the services for an HIV test (4.7%), and miscarriage/post-abortion care (2.8%). For the service location, more participants used the private clinics (54.9%) compared to the clinic in the IZ (36.3%) and commune health center (17.2%). Married women were significantly more likely to use the SRH/HIV services than single women (*p* < 0.001).

### Factors Associated With Condom Use at Last Sex for All Women

Table 4 presents the results of multivariate logistic regression analysis for all participants. Single participants were over 4 times more likely to use condoms at last sex compared to married participants (OR = 4.67; 95%CI = 2.96–7.85). Those with vocational school or higher education was more likely to use condom (OR = 2.19; 95%CI = 1.05–4.57). However, neither HIV knowledge or alcohol use were associated with condom use at last sex.

**Table 4:** Multivariate analysis of factors associated with condom use at last sex.

	**Condom use at last sex**		
	**aOR^**a**^**	**95%CI**	***P***
**Age (years)**			
18–24	1		
25–29	1.33	0.83–2.13	0.236
**Education**			
Secondary			
High school	1.67	0.97–2.86	0.055
Vocational school/College or higher	2.19	1.05–4.57	0.036
**Ethnicity**			
Other	1		
Kinh	1.53	1.01–2.32	0.044
**Marital status**			
Married	1		
Single	4.67	2.78–7.84	0.000
**Residence**			
Dormitory	**1**		
Rent cluster	1.13	0.64–2.01	0.657
**Use of SHR/HIV services while living in the IZ**	0.88		
**Knowledge of HIV/AIDS**	1.02	0.91–1.14	0.315
**Perceived risk of HIV infection**	1.68	0.60–4.75	0.320
**Alcohol use**			
Low risk	1		
Moderate risk	0.64	0.25–1.65	0.359
High risk	1.01	0.14–7.24	0.991

a *Adjusted Odd Ratio*.

## Discussion

The main findings from this study are that (1) single participants reported high rates of first sex while living in the IZ and high rates of condom use during their first time sex, however, they had low levels of condom use at last sex, (2) the majority of single and married participants had sex with only one partner, (3) while the majority of married participants used the SRH/HIV services since living in the IZ, nearly 80% of the single participants had never used SRH/HIV services since living in the IZ and (4) HIV was almost non-existent in this subpopulation. There can be several possible explanations for low HIV prevalence among this group. First, Vietnam has done an effective job in reducing HIV among the general population including IZ workers in the country; secondly these migrants may have participated in HIV intervention projects implemented by the local and international NGOs in the IZ in the past as both the married and single women reported high levels of HIV knowledge. Finally, the positive efforts for HIV education, prevention, and treatment programs in the country could have resulted in a low level of the HIV prevalence despite risk behavior.

It could be argued that those who opted out of the HIV test might include more HIV positives. While age and residence showed differences, sexual behavior, use of SRH services, knowledge and perceptions of risk showed no difference leading us to suggest that levels of HIV were also low among those who opted to not take the test. While HIV was almost non-existent among young women workers, there is a significant subsector that is at greater sexual risk and need the kind of services that can ensure that HIV prevalence remains low. Of the ~28.3% who had sex, 59% of the single participants reported having first sexual intercourse while living in the IZ which is much higher than the rates (12.6%) in another recent study among women migrants in Vietnam (44). Premarital sex in this study is also much higher than in two other comparable studies among unmarried female migrant workers in Shanghai (45) and Heifer, China (8) which found that 28–35% reported premarital sex for the first time in the IZ. While the rates of premarital sex were low in Vietnam in the past (46), the rapid changes in social and economic development and urbanization have resulted in greater openness and more liberal attitudes sexual behavior among young people, with premarital sex become increasingly common among Vietnamese young people (47). In addition, migrants are less constrained by local rural norms regarding sexual behaviors, and they are therefore more likely to feel free to engage in sexual behaviors. A higher level of premarital sex among this population suggests the urgent need to develop programs to promote safer sex among young migrants.

Among the single participants who reported having sexual intercourse, 61.5% had not used condoms or inconsistently used them when having sex in the previous 6 months. The combination of premarital sex and low level of consistent condom use could place these women at risk of sexually transmitted diseases as well as unwanted pregnancy and abortion even if their primary partner was their boyfriend or fiancé. There may be several reasons contributing to the inconsistent condom use among this population. One of the reasons could be that women may not always be the ones to make decision about condom use when having sex. Traditional gender norms make it difficult for Vietnamese women to communicate with male partners about sexual behaviors (e.g., condom use) as they are supposed to be submissive in sexual activities (48). For example, findings of a study among unmarried women seeking abortion in Ho Chi Minh city in Vietnam showed that premarital sex was viewed as a commitment to maintain a relationship with boyfriends who also have strong influence on using contraceptives including condom use (49). Therefore, it is essential for SRH and HIV education and intervention programs addressing safe sexual behaviors engage both young women and young men in the programs.

Unlike findings from a recent study among male and female migrants in the other IZs in Vietnam showing that those who were married and living with partners were more likely to use condoms than single participants (5), our findings documented that unmarried women were more likely to use condoms at the last sex intercourse than those ever married. It is possible that those who are married were less likely to use condoms because they had sex with the husbands who were visiting them in the IZ or while they were visiting husbands in the rural areas. On the other hand, single participants may have been worried about becoming pregnant or contracting STIs/HIV when having sex with their boyfriends and therefore, were more likely use condoms.

Only 20.1% of the women who were single reported ever utilizing SRH/HIV services while living and working in the IZ. Notable is that the use of SRH/HIV services while living in the IZ was significantly associated with condom use at last intercourse. It is likely that those who used the SRH/HIV services received counseling and more information on SRH and therefore were more likely to use condoms. A study among single female migrants in Hanoi found that unlike married migrants, fewer unmarried female migrants went to have a gynecological examination due to shyness (50). Furthermore, SRH programs for unmarried young people is not given a priority in Vietnam, resulting in limited RH services available for unmarried young people (35). This finding provides evidence supporting the need to develop interventions providing culturally appropriate SRH education and reducing stigma and shame for unmarried young migrants to help them improve SRH knowledge as well as access to SRH services which in turn prevent outcomes related to risky sexual behaviors.

### Limitations

Our study has some limitations. Given the nature of the cross-sectional design, our findings should not be interpreted beyond associations, and the role of causality may not be inferred. Generalization of the findings to other migrant populations cannot be made as female migrant workers were recruited from one IZ which is not representative of IZs in Vietnam. Our study only recruited female migrant workers and so the HIV prevalence among male migrant workers could be different. Only 60% of the participants were consented to take HIV and 40% refused; and therefore, we do not know HIV status among those who did not consent. Participants were given incentive to compensate for their time in the survey. However, the incentive could attract more respondents who participated in the survey only for the incentive and may also quickly respond to the survey instead of giving thoughtful responses. Nevertheless, these limitations are generally outweighed by the study's strengths including the first study examining HIV prevalence among migrants working in the IZ, a large sample size and assessment of several sexual risk behaviors for unmarried and married female migrant workers.

## Conclusion

This study indicated that HIV prevalence is very low among young women workers in the IZ in Vietnam. Nonetheless, this study documented a significant subsector in which a number of risky sexual behavior and low levels of condom use at last sex and a low level of using SRH/HIV among young unmarried women workers in the IZ and highlights a need to develop SRH/HIV programs targeting female migrants to raise their awareness about the importance of using SRH/HIV services. The study provides the basis for identifying women at risk and providing them with education, service access and condoms to keep them safe while they do essential work in support of their future, their families and the country.

## Data Availability Statement

The raw data supporting the conclusions of this article will be made available by the authors, without undue reservation.

## Ethics Statement

The studies involving human participants were reviewed and approved by the Institutional Review Boards of the University of Connecticut Health Center, USA and the Institute of Social and Medical Studies, Hanoi, Vietnam. The patients/participants provided their written informed consent to participate in this study.

## Author Contributions

TH, SS, and JS drafted the manuscript and conducted a literature review. TN and NN contributed to data collection. TH and TN performed the statistical analysis. JS and NN reviewed a draft and provided comments for the manuscript. All authors reviewed a manuscript and approved the final draft.

## Funding

This work received funding from the National Institute of Mental Health/National Institutes of Health, USA under the award number 1R21MH118986-01A1.

## Conflict of Interest

The authors declare that the research was conducted in the absence of any commercial or financial relationships that could be construed as a potential conflict of interest.

## Publisher's Note

All claims expressed in this article are solely those of the authors and do not necessarily represent those of their affiliated organizations, or those of the publisher, the editors and the reviewers. Any product that may be evaluated in this article, or claim that may be made by its manufacturer, is not guaranteed or endorsed by the publisher.
